# Effect of spray-drying and ultraviolet C radiation as biosafety steps for CSFV and ASFV inactivation in porcine plasma

**DOI:** 10.1371/journal.pone.0249935

**Published:** 2021-04-28

**Authors:** Elena Blázquez, Carmen Rodríguez, Jesús Ródenas, Rosa Rosell, Joaquim Segalés, Joan Pujols, Javier Polo

**Affiliations:** 1 R&D Department, APC EUROPE, S.L.U., Granollers, Barcelona, Spain; 2 IRTA, Centre de Recerca en Sanitat Animal (CReSA, IRTA-UAB), Campus de la Universitat Autònoma de Barcelona, Bellaterra, Barcelona, Spain; 3 Departament d’Agricultura, Ramaderia, Pesca i Alimentació (DARP) Generalitat de Catalunya, Campus de la Universitat Autònoma de Barcelona, Bellaterra, Barcelona, Spain; 4 OIE Collaborating Centre for the Research and Control of Emerging and Re-emerging Swine Diseases in Europe (IRTA-CReSA), Bellaterra, Barcelona, Spain; 5 Departament de Sanitat i Anatomia Animals, Universitat Autònoma de Barcelona (UAB), Bellaterra, Barcelona, Spain; 6 UAB, Centre de Recerca en Sanitat Animal (CReSA, IRTA-UAB), Campus de la Universitat Autònoma de Barcelona, 08193 Bellaterra, Barcelona, Spain; 7 R&D Department, APC LLC, Ankeny, IA, United States of America; Plum Island Animal Disease Center, UNITED STATES

## Abstract

Spray-dried animal plasma (SDAP) is widely used in diets of domestic animals to improve health status and increase growth and feed efficiency. Individual steps in the SDAP manufacturing process, including spray-drying, have been validated to inactivate potential pathogens. Manufacturing standards have established a minimum exit temperature of 80°C and a minimum post-drying storage period of 14 days at 20°C for production of SDAP. Also, UV-C irradiation has been evaluated as another inactivation step that could be included in the manufacturing process. The aim of this study was to assess the inactivation effectiveness of spray-drying on Classical swine fever virus (CSFV) and African swine fever virus (ASFV) and the effect of UV-C inactivation on ASFV as redundant biosafety steps of the manufacturing process for producing spray-dried porcine plasma (SDPP). This study demonstrated that UV-C treatment of liquid porcine plasma can inactivate more than 4 Log_10_ TCID_50_/mL of ASFV at 3000 J/L. Spray-drying effectively inactivated at least 4 Log_10_ TCID_50_/mL of both CSFV and ASFV. Incorporating UV-C technology within the SDAP manufacturing process can add another biosafety step to further enhance product safety.

## Introduction

Spray-dried animal plasma (SDAP) is a natural co-product of the meat packing industry that is widely used in swine diets to improve health status and increase growth and feed efficiency [1, 2]. Blood is harvested from animals inspected and passed as fit for slaughter for human consumption. Blood is collected into containers with anticoagulant, chilled, and centrifuged to separate plasma. Liquid plasma is then concentrated and spray-dried to produce ingredients used in food, feed and industrial applications [3].

Individual steps in the SDAP manufacturing process have been validated to inactivate potential pathogens [4]. Globally, most plasma producers have adapted processing standards recommended by the European Animal Protein Association (EAPA Code of Practice, https://www.eapa.biz/quality-safety). Briefly, these standards have established a minimum exit temperature of 80°C and a minimum post-drying storage period of 14 days at 20°C. Other inactivation steps, such as UV-C irradiation of liquid plasma, have been evaluated that could be included in the manufacturing process [4].

The World Health Organization (WHO) has published guidelines for the manufacture of human blood products [5]. The WHO recommends that the manufacturing process for human blood products should include one or two robust processing steps that can inactivate non-enveloped or enveloped viruses. A robust processing step is defined as one that can inactivate 4 Log of virus titer [5].

Ultraviolet-C (UV-C) light is a shortwave electromagnetic radiation with a wavelength of 254 nm (range of 250 and 270 nm) that induces damage in nucleic acids by disrupting DNA or RNA structure [6]. UV-C technology has been used to disinfect water, food products and surfaces [7, 8], and is an alternative to chemical inactivation methods [9]. Previous studies demonstrated that UV-C was effective for inactivating substantial amounts of enveloped and non-enveloped swine viruses in liquid plasma [10].

Classical swine fever virus (CSFV) is an enveloped +ssRNA virus of the *Flaviviridae* family, and is considered a virus that has low survival when exposed to high temperatures [11]. African swine fever virus (ASFV) is an enveloped dsDNA virus belonging to the *Asfarviridae* family [12] that can cause high mortality in pigs of all ages and is considered resistant to high temperatures [13]. Both viruses affect swine and are notifiable diseases to the World Organization for Animal Health (OIE) [11].

The aim of this study was to assess inactivation efficiency of spray-drying on CSFV and ASFV and UV-C inactivation on ASFV to evaluate these processes as redundant biosafety steps in the manufacturing process for producing spray-dried porcine plasma (SDPP).

## Material and methods

### Virus and cells

#### Classical swine fever virus

The CSFV strain Alfort-187 and the PK-15 cell line was provided by the EU Reference Laboratory for CSF, Institute of Virology, Hanover, Germany. The CSFV was propagated in the PK-15 cell line and grown in modified Eagle medium (MEM) that was supplemented with 5% pestivirus antibody negative fetal bovine serum (FBS), 200 mM glutamine, 100 UI penicillin /mL, 100 μg streptomycin /mL and 40 UI nystatin /mL). CSFV viral stock was produced in successive passages on PK-15 cell line until a final viral titer of 10^7.5^ TCID_50_ /mL was acheived. Titration of viral stock solution was done on PK-15 cells following the OIE CSFV immunoperoxidase technique (IPT) standard protocol [14].

#### African swine fever virus

The ASFV strain Badajoz-71 was adapted to Vero cells (ASFV-BA71-V) [15] and was provided by Dr. María Luisa Salas from Centro de Biología Molecular Severo Ochoa (CBMSO), Madrid, Spain. The virus was propagated in Vero cells (ATCC CCL-81) grown in DMEM supplemented with 10% FBS, 200 mM glutamine, 100 UI penicillin /mL, 100 μg streptomycin /mL, and 40 UI nystatin /mL. The final stock solution was titrated following the IPT OIE standard protocol for ASFV [16] and a final ASFV titer of 10^6.9^ TCID_50_ /mL was achieved.

### Plasma

The plasma used to determine ASFV inactivation by UV and CSFV inactivation by spray- drying was obtained in EU porcine slaughter facilities from animals inspected and approved for slaughter for human consumption. Blood was collected in stainless steel containers, with an anticoagulant, refrigerated and transported to the APC Europe facilities (APC-Europe S.L.U., Granollers, Spain). Plasma was separated by commercial centrifugation and frozen at -20°C until use.

The plasma used to determine ASFV inactivation by spray-drying was collected as described above but had been commercially spray dried. A total of 2.7 kg of commercial SDP (Appetein GS; APC Europe S.L.U.) was treated with 10 kGy ionizing radiation (Aragogamma, S.A. Les Franqueses del Vallés, Spain). Ten kGy ionizing radiation was sufficient to sterilize the SDP and prevent potential bacterial interference during the virus titration in Vero cells. After kGy radiation, the SDP was solubilized in sterile water to achieve 28% solids. A 10-mL sample of solubilized plasma was stored at –80°C and later tested for the presence of ASFV antibodies (INgezim PPA COMPAC, INGENASA; Madrid, Spain) or genome [17].

### Spray-drying: Inactivation of ASFV and CSFV

Three 1-L batches of unconcentrated porcine plasma were thawed, filtered, and a 10 mL sample of each batch was stored at –80°C for later analysis. A volume of 0.010 L of CSFV stock solution (10^7.5^ TCID_50_/mL) was added to 0.990 L of plasma to obtain a theoretical final viral titer of 10^5.5^ TCID_50_/mL.

For the ASFV experiment, each of three 1-L batches of the reconstituted (28% solids) irradiated SDP were sampled. A 0.1 L sample was stored at –80°C for later analysis and the remaining 0.9 L w14as inoculated with 0.1 L of ASFV stock solution (10^6.9^ TCID_50_/mL) to achieve a viral titer of approximately 10^5.9^ TCID_50_/mL in each 1-L batch.

The laboratory spray-drier (Büchi Mini Spray Dryer B-290, Büchi Labortechnik, Switzerland) used in these experiments was adjusted to an inlet air temperature of 200 ± 5°C and an outlet air temperature set at 80 ± 1°C as previously described [18]. Airflow through the column and the suspension flow to the nozzle was set at 45 m_3_ h^-1^ (at 20°C) and 0.2 lh^-1^, respectively. The airflow through the feed nozzle was adjusted to 0.7 m^3^h^-1^ (at 20°C). Residence time was estimated to be 0.41 s.

Plasma samples were collected after spray-drying to know the inactivation effect of the laboratory spray-drier process alone. Then sub-samples of spray-dried product collected after spray-drying were kept at 80°C for 60 s to simulate the typical residence time of the dried product in a commercial spray drier [19]. Triplicate 0.5 g samples of spray-dried plasma kept in 0.5 cm glass tubes (inner diameter) were placed in a water bath set at 90±1°C for 60 s. The 90°C water bath temperature was necessary to assure that the spray dried plasma in the tubes was maintained at 80°C inside the tube. Thermal probes were used to monitor the temperature of the spray dried plasma in the tubes and to assure that the temperature of the dried plasma was maintained at the desired 80°C for 60 s. Samples were then placed in a dry-ice cooled container until all samples were processed, then stored at -80°C until final analysis.

Prior to viral titration, the spray-dried samples were reconstituted by adding 5.5 mL of distilled water to 0.5 g of plasma sample. Titration of virus was done using the whole plate for each dilution (from -1 to -5 dilutions using 12- well plates) to amplify the detection capability of the test. Plates were read by IPT following the OIE standard protocol for ASFV [16] available in https://asf-referencelab.info/asf/images/ficherosasf/SOP%202018/SOP-ASF-IPT-1_REV2018.pdf and in the case of CSFV, the EU diagnostic manual for CSFV was followed [20]. In both cases, plates were quantified by the Reed and Muench procedure [21].

Negative samples in the titration assay were passaged in a blinded fashion to detect very low quantities of CSFV or ASFV by inoculating 50 mL of the reconstituted post-dried samples on 10 different 175 cm^2^ flasks containing either PK-15 cells for CSFV or Vero cells for ASFV (5 mL reconstituted dried sample for each 175 cm^2^ flask). After three to four days, cell cultures were harvested and passed to new PK-15 or Vero cell cultures, respectively. Three serial passages and virus screening by IPT were done before a sample was considered negative.

The reduction factor was calculated as the difference between the virus titer detected in the inoculated material at start and the titer detected in the final sample after processing.

### UV-C: Inactivation of ASFV

A total of 22 L of unconcentrated porcine plasma was inoculated with 2 L of stock viral solution (containing 10^6.8^ TCID_50_/mL) achieving an approximate virus titre of 10^5.7^ TCID_50_/mL. The 24 L of inoculated plasma was sub-divided into three 8 L batches. The operation time of the UV-C treatment is based on the quantity of product to be irradiated and the flow rate of the product feed. At a flow rate of 4000 L/h, 9 s are required for 10 L of product to pass through the reactor once; thus, one turn of the product through the system is equivalent to a UV-C dose of 22.95 J/L. The UV-C dosage is expressed as J/L.

The flow of the inoculated plasma was stabilized at 4000 L/h with the UV lamp switched off. After 5 minutes of stable flow, a positive control (0 J/L) sample was collected into a sterile container. Then, the UV-C lamp was switched on and irradiation started. A volume of 175 mL of treated plasma was collected into sterile containers at different UV-C doses (750, 1500, 3000, 6000, and 9000 J/L) and stored at -80°C for subsequent analysis.

The UV-C reactor system SurePure Turbulator^TM^ SP-1 was manufactured by SurePure Operation AG (Zug, Switzerland) and has been previously described [10].

A standard ‘Cleaning in Place’ (CIP) process based on a treatment with 5% NaOH, was implemented before and after each UV-C treatment [22].

Quantification of ASFV in plasma samples was analyzed by titration on Vero CCL81 cell culture using the microtiter assay procedure [23]. Titration of virus was done as previously described.

### Modeling of inactivation

Microbial inactivation kinetics models have been previously described [24]. The GInaFiT software includes eight common models describing linear and non-linear inactivation curves [24].

GInaFiT software determines the goodness of fit in terms of root mean square error (RMSE) for all the tested models. The mathematical model that presents the lowest RMSE value is considered the model that best fits the data.

The equations that describe the different mathematical models used in this study are detailed below:

The biphasic model [25] uses the Eq (1):

$$log10(N)=log10(N0)+log10(f*e^{{-k}_{max1}t}+(1-f)*e^{{-k}_{max2}t})$$ (1)

The Biphasic plus shoulder model [24] follows the Eq (2):

$$log10(N)=log10\;(N0)+log10\;\left(f*\exp(-\mathrm{kmax}1*t)*\frac{\exp(\mathrm{kmax}1*\mathrm{Sl})}{1+(\exp(\mathrm{kmax}1*\mathrm{Sl})-1*\exp(-\mathrm{kmax}1*t))+1(1-f)*\exp(-\mathrm{kmax}2*t)*\frac{\exp(\mathrm{kmax}1*\mathrm{Sl})}{{\left(1+(\exp(\mathrm{kmax}1*\mathrm{Sl})-1)*\exp(-\mathrm{kmax}1*t)\right)}^{\frac{\mathrm{kmax}2}{\mathrm{kmax}1}}}}\right)$$ (2)

Where *N0* is the initial bacterial concentration; *t* is time; *k*_*max1*_ and *k*_*max2*_ are the specific inactivation rates of the two populations and *S1* are the degrees of freedom used for the parameter estimation by GInaFiT.

The Weibull model [26] uses the Eq (3):

$$log10(N)=log10(N(0))-(\frac{t}{\delta }{)}^{p}$$ (3)

Where *N* represents the microbial cell density, *N0* the initial microbial cell density, *t* is time, *δ* is a scale parameter denoted as the time for the first decimal reduction, and *p* is the shape parameter that describes concavity or convexity of the curve. The curve shows convexity if p>1 and the curve is concave if p<1.

Data were expressed as the mean Log_10_ TCID_50_ with the standard deviation of three replicates. Mean square error (MSE), goodness of fit, correlation coefficient (R^2^), adjusted correlation coefficient (adj-R^2^) was calculated, and 4D values (the dose needed to inactivate 4 Log_10_ of viral load) were estimated.

## Results

The original plasma used in these experiments was confirmed to be negative for antibodies and genome for the viruses tested.

### Spray-drying experiment

Viral inactivation by spray drying is summarized in Table 1. The laboratory spray drying process alone had a residence time < 1 sec and a reduction factor of 2.06 and 2.11 Log_10_ TCID_50_/mL for CSFV and ASFV, respectively. However, after the residence time simulation of the conditions in commercial driers at 80°C for 60 s a reduction factor of 4.11 and 5.78 Log_10_ TCID_50_/mL was achieved for ASFV and CSFV respectively. CSFV negative samples were subjected to three successive blind passages before being declared negative.

**Table 1:** Titration of CSFV and ASFV samples before and after the spray drying treatment (Mean ± SD).

	CSFV		ASFV	
TREATMENT	MEAN VIRUS RECOVERED LOG_10_ TCID_50_/mL	REDUCTION FACTOR (RF)	MEAN VIRUS RECOVERED LOG_10_ TCID_50_/mL	REDUCTION FACTOR (RF)
**POSITIVE CONTROL INOCULATED PLASMA**	5.78 ± 0.16	NA^2^	5.77 ± 0.20	NA^2^
**SD 200–80**	3.72 ± 0.20	2.06 ± 0.25	3.66 ± 0.0	2.11 ± 0.20
**SD 200–80 + 60 s 80°C**	NEG^1^	≥5.78	1.66 ± 0.0	4.11 ± 0.20

^(1)^ NEG: negative sample

^(2)^ NA.: does not apply.

### UV-C experiment

**Fig 1:**
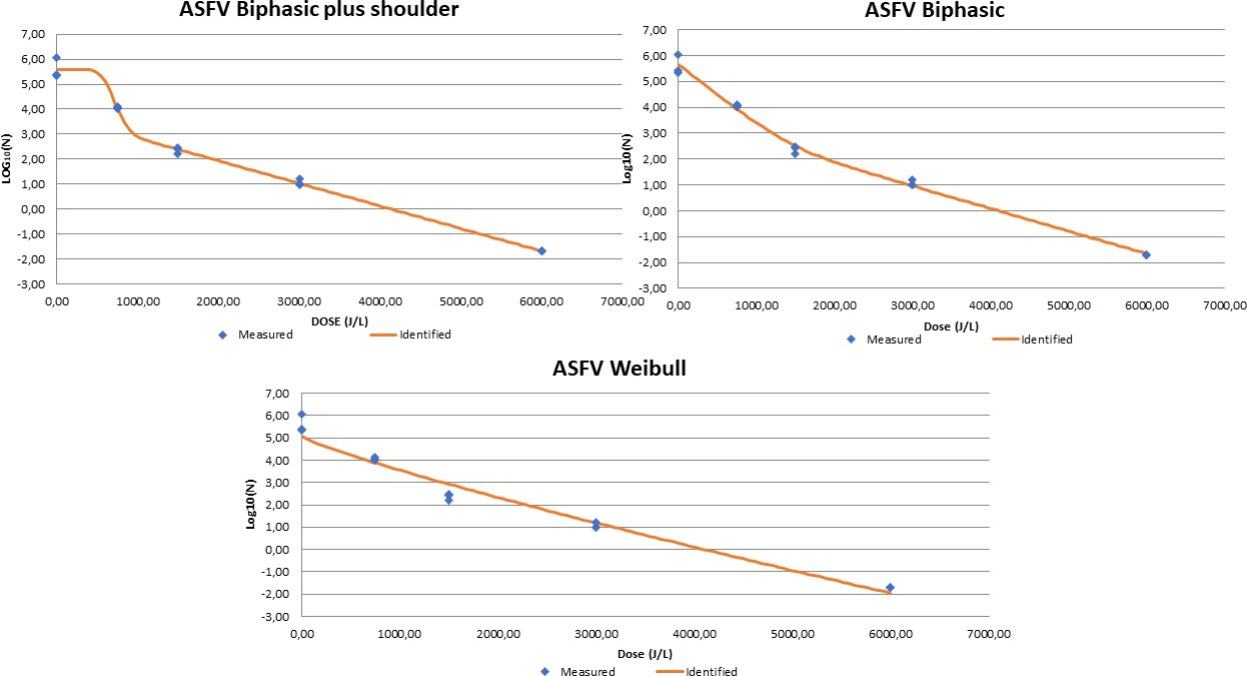
Mean ASFV Log10/mL values after UV-C irradiation of porcine plasma at different UV irradiation doses. Best fit mathematical models are shown. Blue diamonds indicated measured results of the viral titer at different UV-C irradiation doses expressed as mean log 10/mL (n = 3 replicates). Red line is the identified inactivation curve model.

UV-C inactivation results for ASFV are summarized in Table 2 and Fig 1. Negative samples at 6000 J/L for ASFV were subjected to 3 blind passages before being considered negative.

**Table 2:** Titration of ASFV samples subjected to different doses of UV-C irradiation.

UV Dose (J/L)	Mean virus recovered Log_10_ TCID_50_/mL	SD	Accumulated Total Log_10_ reduction
0	5.60	0.39	0.00
750	4.06	0.05	1.54
1500	2.37	0.13	3.23
3000	0.98	0.02	4.62
6000	BDL*	0.00	5.60
9000	BDL*	0.00	NC†

BDL*: Below Detection Limit; NC†: Not Calculated.

The model that best fit the inactivation ASFV data with the lowest RMSE was the biphasic + shoulder (Table 3). The simpler models, including the biphasic and Weibull models, had a slightly greater RMSE and slightly lower correlation coefficient. The more complex models, biphasic + shoulder and the biphasic, describe multiple virus populations with different inactivation kinetics while the simpler Weibull model describes a more uniform viral population with consistent inactivation kinetics. However, all three models resulted in similar 4D value estimates between 2130 and 2239 J/L.

**Table 3:** Kinetics parameters obtained in the three mathematical models with best fit for UV-C inactivation of ASFV.

	Biphasic + shoulder	Biphasic	Weibull
Mean Sum of Squared Error	0.0385	0.0445	0.0714
Root Mean Sum of Squared Error	**0.1963**	0.211	0.2673
R-Square	0.9959	0.9948	0.9909
R-Square, adjusted	0.9943	0.9934	0.9894
4D reduction is reached at (minutes)	±11.71	±11.14	±11.71
4D reduction is reached at (UV J/L)	**2239.54**	2130.525	2239.54

## Discussion

Spray-drying as an intrinsic safety step in the manufacturing process of SDPP has been shown effective to inactivate different viruses of concern for the swine industry including porcine respiratory and reproductive syndrome virus (PRRSV), pseudorabies virus (PRV), swine vesicular disease virus and porcine epidemic diarrhea virus (PEDV) [18, 27–29] as well as bacteria such as *Escherichia coli* or *Salmonella enterica* [30, 31]. Pathogen inactivation by spray drying occurs due to the combination of rapid desiccation at high temperature [19], that results in damage to the cytoplasmic membrane [32, 33], damage to genetic material and inactivation of other proteins including enzymes [33]. During the spray-drying process plasma is exposed to a minimum of 80°C throughout substance [3], which is a temperature recognized as effective to inactivate pathogens such as ASFV, CSFV, Swine vesicular disease virus (SVDV) and Foot and mouth disease virus (FMDV) in cooked meat products [34]. In the present experiments, just the spray-drying process without extended residence time showed a reduction factor of 2.06 and 2.11 Log_10_ TCID_50_/mL for CSFV and ASFV, respectively. These results demonstrate that both viruses were inactivated similarly due to damage caused by rapid dehydration at 80°C associated with a lab drier with a residence time of less than 1 s [19]. However, the spray-dried samples that were subjected to the extended residence time (80°C for 60 s) associated with commercial driers had an inactivation of 4.11 Log_10_ TCID_50_/mL for ASFV and 5.78 Log_10_ TCID_50_/mL for CSFV. Complete inactivation was achieved for CSFV using the extended dwell time (60 s). Spray-drying with 60 s dwell time was slightly more effective for inactivating CSFV suggesting that ASFV is more heat resistant than CSFV. These results agree with others [35, 36] demonstrating that ASFV is more heat stable than CSFV.

The commercial process of SDPP involves several biosafety steps, including collection of blood from healthy animals declared fit for slaughter for human consumption, which excludes collection of blood from sick animals. Therefore, it is unlikely SDPP producers would receive blood from animals with peak viremia. According to the risk assessment conducted by the French safety agency [37], it is very unlikely that a pig at the peak viremia would be accepted for slaughter for human consumption due to the obvious symptoms and bad physical condition of the animal. Therefore, the window of time for an infected asymptomatic ASFV animal to be accepted for slaughter for human consumption would be only a few days (1 to 3) after infection when the amount of virus in the blood would be significantly lower than in viremic animal. In addition, the manufacturing process involved in commercial plasma involves other safety steps. The commercial SDPP process includes a dilution factor for any potential viral load because of the pooling of blood from thousands of clinically healthy animals into silos at the abattoir or at the manufacturing facilities. Therefore, the inactivation of 4.11 Log_10_/mL found in our study can be considered safe for inactivation of ASFV. Furthermore, the manufacturing process of SDPP in US and EU includes the step of storage of the final packed spray-dried product at 20°C for 14 days [4]. Recently Fischer et al. [38], demonstrated that ASFV inoculated on SDPP and stored at room temperature (20°C) for 14 days was inactivated by more than 5 logs. The combination of these various process control steps reduces the risk of ASFV transmission through SDPP to essentially zero, as already recognized by ANSES [37].

UV-C has been widely used for the disinfection of surfaces, water and food products [7, 8, 39, 40] due to its germicidal action. Previous research has demonstrated that UV-C treatment of liquid plasma was effective to inactivate several enveloped and non-enveloped viruses [10, 41] and bacteria [31, 42]. The present results show that UV-C inactivated more than 4 Log of ASFV and agree with previous research suggesting that UV-C is very effective for inactivating enveloped viruses, including CSFV as well. Virucidal effects of UV-C may be associated with damage of the virus genome [6, 43], lipid peroxidation resulting in damage of the envelope membrane of these viruses [10] and cross-linking of nucleocapside proteins [44].

Estimates of UV-C 4D inactivation for a number of viruses range between 1004 to 3708 J/L [10], suggesting some differences in susceptibility to UV-C between virus. In the current experiment, the UV-C 4D value estimate for ASFV was 1912 J/L and is within the range of previously reported data. Blázquez et al., [10] found that the UV-C 4D value for CSFV inactivation was 1641 J/L, slightly below the present estimation for ASFV suggesting that CSFV is somewhat less resistant to UV-C irradiation than ASFV. Biphasic inactivation curves can be explained by a number of phenomena including the cumulative effect of continued damage to the genetic material [44], the presence of various subpopulations differing in susceptibility to UV-C or the protective effect of virus aggregates including viral aggregates with other material [45, 46]. In the present experiment, the best fit model included a biphasic curve with a shoulder. While the Weibull model had a slightly larger RMSE, the estimated 4D value was like that of the biphasic plus shoulder model and the more linear Weibull. Similarly, the best fit models for CSFV UV-C inactivation were the biphasic curve with a shoulder and the Weibull model [10].

The WHO proposed minimum manufacturing standards to assure viral safety for international trade of human plasma products [5]. These standards recommend that the manufacturing process include one or two independent robust safety steps to inactivate a non-enveloped or enveloped virus, respectively. They define a robust safety step as one capable of inactivating 4 log of virus. In addition, the WHO recognize that UV-C can inactivate a wide range of organisms including viruses. Therefore, the data in the present work demonstrate that both, the UV-C irradiation at 3000 J/L and the spray drying process achieving 80°C throughout its substance, are two independent safety steps that meets the WHO standard for a recognition of a robust processing step. Spray drying and UV-C inactivate viruses by different mechanisms and the potential synergistic effect of the combined methods may result to a higher degree of inactivation, but this has not been tested.

In conclusion, this study demonstrated that spray-drying as an independent treatment is very effective at inactivating both CSFV and ASFV, achieving a reduction of 4 Log_10_ TCID_50_/mL. Furthermore, UV-C treatment of liquid porcine plasma can inactivate more than 4 Log_10_ TCID_50_/mL of ASFV at 3000 J/L. Thus, incorporating UV-C technology with the traditional SDP manufacturing process can add a redundant biosafety step to further enhance product safety.
